# Chromaticity-Tunable and Thermal Stable Phosphor-in-Glass Inorganic Color Converter for High Power Warm w-LEDs

**DOI:** 10.3390/ma11101792

**Published:** 2018-09-21

**Authors:** Zikun Chen, Bo Wang, Xiaoshuang Li, Dayu Huang, Hongyang Sun, Qingguang Zeng

**Affiliations:** School of Applied Physics and Materials, Wuyi University, Jiangmen 529020, China; chenzk1993@163.com (Z.C.); lixiaoshuang12@mails.ucas.ac.cn (X.L.); dyhuang@ciac.ac.cn (D.H.); 15667096212@163.com (H.S.)

**Keywords:** garnet, luminescent property, phosphor-in-glass, thermal stability

## Abstract

In this work, an aluminate silicate garnet phosphor, Y_2_Mg_2_Al_2_Si_2_O_12_:Ce^3+^ (YMASG:Ce^3+^), exhibiting strong and broad yellow-orange emission, was successfully synthesized. Attributed to the double cation substitution of YAG:Ce^3+^, which led to a compression effect, a redshift was observed with respect to YAG:Ce^3+^. More importantly, a transparent phosphor-in-glass (PiG) sample was obtained by incorporating the phosphor YMASG:Ce^3+^ into a special low-melting precursor glass. The energy dispersive spectrometer (EDS) mapping analysis of the as-prepared PiG sample indicates that YMASG:Ce^3+^ was successfully incorporated into the glass host, and its powders were uniformly distributed in glass. The photoluminescence intensity of the PiG sample was higher than that of the powder due to its relatively high thermal conductivity. Additionally, the combination of the PiG sample and a blue high-power chip generated a modular white LED with a luminous efficacy of 54.5 lm/W, a correlated color temperature (CCT) of 5274 K, and a color rendering index (CRI) of 79.5 at 350 mA.

## 1. Introduction

In past decades, scientists and engineers have been interested in white light-emitting diodes (w-LEDs) because of their extremely long life and high energy efficiency relative to conventional lighting sources [1,2,3]. Despite the fast progress in developing LED technology, mainstream LED products that combine blue LEDs with a mixture of Y_3_Al_5_O_12_:Ce^3+^ (YAG:Ce^3+^) and organic encapsulation materials still suffer significant technical problems of shifting on chromaticity and degradation in luminous efficacy (LE) caused by the yellowing and aging of silicone or epoxy resin [4,5,6,7]. Moreover, the drawback of congenitally insufficient red component in YAG:Ce^3+^ gives rise to a high correlated color temperature (CCT) and a low color rendering index (CRI) for the white light, which limits the wider application of w-LEDs in the high-quality indoor lighting field [8,9].

To solve the above problems, an all-inorganic light convertor with excellent thermal stability is of vital importance. Recently, a phosphor-in-glass (PiG) strategy, consisting of dispersing phosphors directly into a low-melting inorganic precursor glass, which exhibits great superiority as an encapsulant over organic resins, was proposed by Lee et al. [10]. Phosphor-in-glass (PiG) has attracted attention for its excellent thermal stability and high thermal conductivity. However, the problem of red deficiency remains, so such white LED devices have been unable to meet the demand for high-quality lighting sources. To improve the CRI, one or more phosphors (green or red) were mixed with YAG:Ce^3+^, and a multicolor emitting PiG was then obtained by incorporating the phosphors into a glass frit. Nevertheless, severe photon reabsorption occurred between the yellow/green and red phosphors, leading to transparency change and luminous efficacy reduction [11,12,13].

Aiming to tune the CCT and CRI of YAG:Ce^3+^ PiG-based LED devices, novel phosphors emitting red-rich light are in demand. In this work, we successfully synthesized a garnet-based phosphor Y_2_Mg_2_Al_2_Si_2_O_12_:Ce^3+^ (YMASG:Ce^3+^) via the double substitution of Mg^2+^–Si^4+^ for Y^3+^–Al^3+^ in the YAG:Ce^3+^ host lattice. This substitution gives rise to the change of the crystal field, following with a redshift in the emission band. A garnet-based PiG was then prepared by incorporating YMASG:Ce^3+^ into a special low-melting precursor glass. X-ray fluorescence and SEM analysis of the as-prepared PiG sample prove that YMASG:Ce^3+^ was successfully incorporated into the glass host and the particles were uniformly distributed in glass. The luminescent property measurements of the PiG sample indicate that the emission intensity at 420 K is retained 53%, and only a slight shift is observed in the photoluminescence (PL) spectrum. A warm w-LEDs device was then obtained using the transparent PiG sample, and the related CCT and CRI of the device are 5274 K and 79.5, respectively, under 350 mA. This study provides a strategy based on local structure correlation to obtain phosphors with good thermal stability for warm w-LEDs.

## 2. Experimental

### 2.1. Sample Preparation

A series of phosphors YMASG:xCe^3+^ (x = 0.01, 0.02, 0.03, 0.04, 0.05, 0.06, 0.07, and 0.09) were synthesized by high-temperature solid state technique. The starting reagents Y_2_O_3_ and CeO_2_ were purchased from Sinopharm Chemical Reagent (Beijing, China), while Al_2_O_3_, SiO_2_, and CeO_2_ were purchased from Beijing Chemical Company (Beijing, China). Mixtures of Y_2_O_3_ (99.9%), MgO (99.9%), Al_2_O_3_ (99.5%), SiO_2_ (99.9%), and CeO_2_ (99.9%) according to the stoichiometric ratio were ground uniformly and loaded into an agate mortar. The mixtures were then fired at 800 °C for 1 h and later sintered at 1400 °C for 6 h in a reducing atmosphere (5%H_2_ + 95%N_2_).

To achieve the luminescent PiG, precursor glasses with following composition (mol %) of 55TeO_2_-7B_2_O_3_-18ZnO-20Na_2_O were prepared by a conventional melting-quenching method. The reagent grade chemicals were mixed thoroughly and melted in an alumina crucible at 780 °C for 30 min in ambient atmosphere. The melt was poured into a cold copper mold and then cooled to room temperature. The as-synthesized glass was then milled to powders and mixed with phosphors and sintered in an alumina crucible at 560 °C for 20 min. The melt was poured into a 230 °C preheated copper mold and cooled to room temperature [7].

### 2.2. Characterizations

X-ray diffraction (XRD) analyses of all the powder samples were performed at room temperature with a powder diffractometer (X’ Pert PRO, Cu Kα, λ = 1.5418 Å, PANalytical, Almelo, The Netherlands). The photoluminescence (PL) and photoluminescence excitation (PLE) spectra of YMASG:xCe^3+^ (x = 0.01, 0.02, 0.03, 0.04, 0.05, 0.06, 0.07, and 0.09) and the PiG sample were recorded by Edinburgh Instruments (FLS980, Livingston, UK) equipped with 450 W xenon lamps. The quantum efficiency (QE) measurements were performed using a barium sulfate coated integrating sphere that attached to the spectrophotometer. The QE, defined as the ratio of the total number of photons emitted (*I_em_*) to the number of photons absorbed (*I_abs_*), is expressed as

$$\eta =\frac{I_{em}}{I_{abs}}=\frac{\int L_{S}}{\int E_{R}-\int E_{S}}\;$$

where *L*_S_ is the emission spectrum of the sample, *E_S_* and *E_R_* are the spectra of the excitation light with and without the sample in the integrating sphere. The morphologies of YMASG:0.05Ce^3+^ and the PiG sample were characterized using a scanning electron microscope (SEM, Zeiss Sigma500, Jena, Germany). The X-ray fluorescence (XRF) analysis was conducted by M4 Tornado (Bruker, Germany). The surface temperature of the PiG sample was measured by a thermographic camera (Testo 882, Testo, Germany). An integrating sphere of 50 cm diameter connected to a charge coupled device (CCD) detector with an optical fiber (HAAS-2000, Everfine Photo-E-Info Co. Ltd., Hangzhou, China) was used to record the electroluminescent (EL) spectra of the obtained warm w-LED device.

## 3. Results and Discussion

### 3.1. Phase Analysis

**Figure 1:**
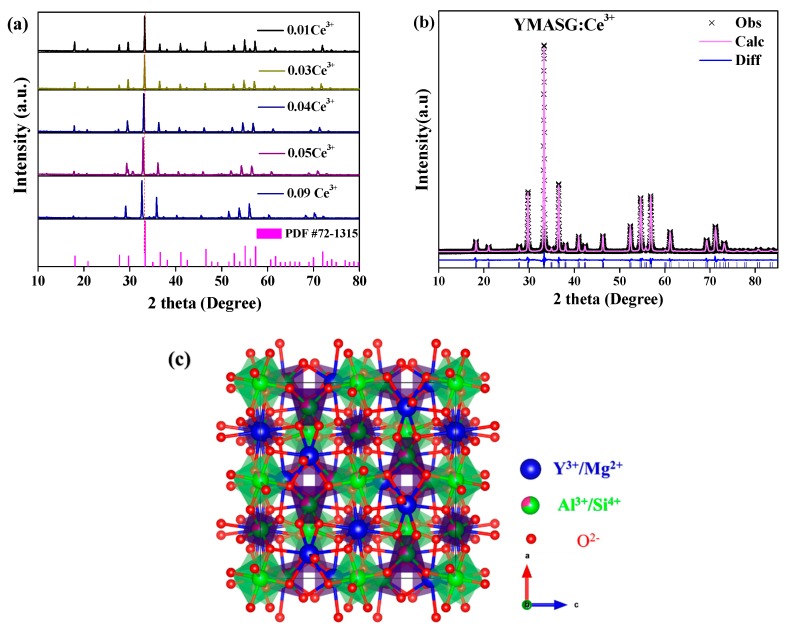
(**a**) The XRD patterns of YMASG:xCe^3+^ (x = 0.01, 0.03, 0.04, 0.05, and 0.09) with the standard pattern of YAG (PDF #72–1315) as a reference. (**b**) The XRD refinement results of YMASG: 0.05Ce^3+^. (**c**) The unit crystal structure of the YMASG host.

The phase purities along with crystal structures of the synthesized YMASG:xCe^3+^ (x = 0.01, 0.03, 0.04, 0.05, and 0.09) were investigated by XRD and Rietveld profile refinements. Since Ce^3+^ ion has an ionic radius comparable to that of Y^3+^ ion, the Ce^3+^ ion should substitute the Y^3+^ ion in YMASG:Ce^3+^. Figure 1a shows the XRD patterns of YMASG:xCe^3+^ (x = 0.01, 0.03, 0.04, 0.05, and 0.09). It is clear that the crystal structures of the samples crystallized in the garnet structure (cubic space group, *Ia3d*). The disappearance of some diffraction peaks in the YMASG:Ce^3+^ patterns may be attributed to the preferential orientation of the samples. In addition, all patterns of the YMASG:xCe^3+^ (x = 0.01, 0.03, 0.04, 0.05, and 0.09) are highly consistent with the pure phase of YAG (PDF #72–1315) [14], and there are no diffraction peaks of impurities, implying that the Ce^3+^ ion doping has not changed the crystal structure significantly. Furthermore, the diffraction peaks gradually shift to lower angles as the doping concentration of Ce^3+^ ion increases because the larger ions Ce^3+^ occupy Y^3+^ sites in the YMASG host lattice.

To verify the crystal structures of the samples, YMASG:0.05Ce^3+^, which exhibits an intense yellow body color, was chosen representatively to perform Rietveld structure refinements with the GSAS program. The starting model was built with crystallographic data known for the YAG structure (ICSD-20090). The refinement pattern of MYASG:0.05Ce^3+^ is displayed in Figure 1b, and the reliability refinement factors of *R*_wp_ and *R*_p_ were determined to be 10.03% and 7.62%, respectively, suggesting that the atom positions, fraction factors, and temperature factors of YMASG:0.05Ce^3+^ all well satisfy the reflection conditions [15]. The final refined crystallographic data and reliability factors for the sample are listed in Table 1. In addition, the results show that the cell volume of YMASG:0.05Ce^3+^ (1702.1 Å^3^) was smaller than that of YAG (1731.5 Å^3^) because of the replacement of the larger Y^3+^/Al^3+^ ions by the smaller Mg^2+^/Si^4+^ ions.

A schematic spatial view of the YMASG structure according to the refinement results is displayed in Figure 1c, from which it can be seen that the network of YMASG is constructed by vertexes sharing octahedra and tetrahedra. The Y/Mg atoms are distributed on the dodecahedral sites, while Al/Si atoms coordinate with four or six oxygen atoms. Notably, the substitution of Y atoms by Mg atoms shrinks the volume of the Y/MgO_8_ dodecahedra in YMASG compared with YO_8_ dodecahedra in YAG. Thus, a red shift of Ce^3+^ emission in YMASG:xCe^3+^ samples is expected when considering that the Ce^3+^ ions mainly occupy the distorted dodecahedral sites.

**Figure 2:**
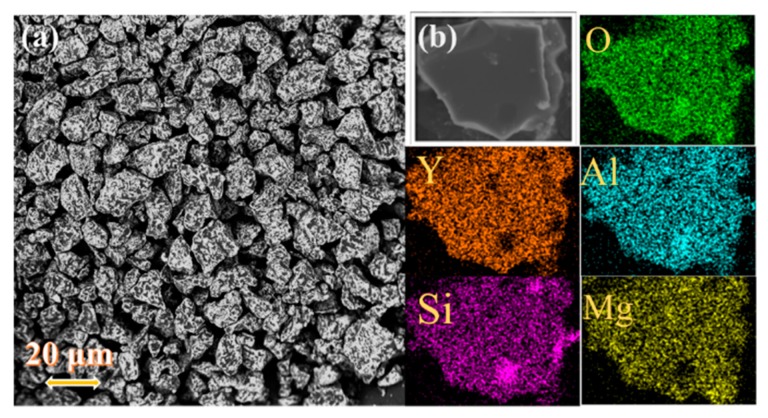
(**a**) The SEM image of YMASG:0.05Ce^3+^. (**b**) The EDS mapping of YMASG:0.05Ce^3+^.

Generally, emission intensity is tied to the morphology, crystallinity, and size of the phosphor particles. In this case, the SEM images of the representative YMASG:0.05Ce^3+^ were measured as shown in Figure 2a. The sample presents an irregular rectangular morphology and the main grain size ranges from 15 to 30 μm. Moreover, the elemental mapping measurement was performed on a typical particle (see Figure 2b). This vividly illustrates that the phosphor comprises the elements Y, Mg, Al, and Si, and the constituent elements are homogeneously distributed in the whole sample without any element aggregation and phase separation.

### 3.2. LuminescentProperties of YMASG:xCe^3+^

**Figure 3:**
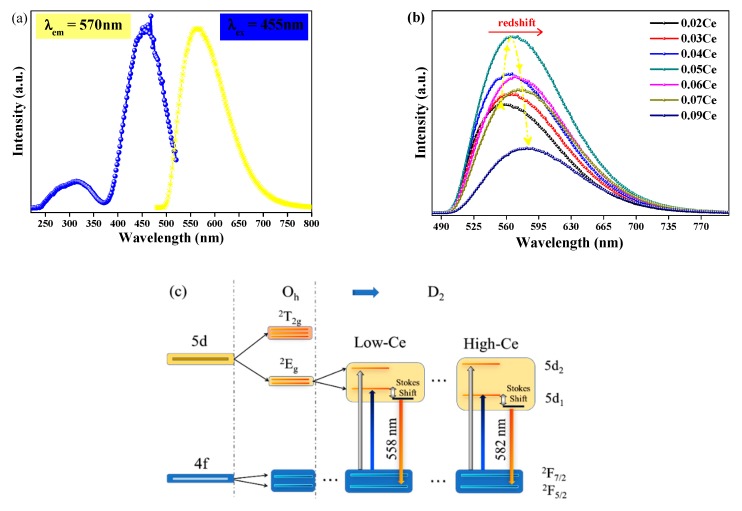
(**a**) The PL (*λ*_ex_ = 455 nm) and PLE (*λ*_em_ = 570 nm) spectra of YMASG:0.05Ce^3+^. (**b**) The PL (*λ*_ex_ = 455 nm) spectra of YMASG:xCe^3+^ (x = 0.01, 0.03, 0.04, 0.05, and 0.09). (**c**) The crystal field splitting and the energy level diagram of Ce^3+^ ion in YMASG during the substitution of Y^3+^/Mg^2+^ ions.

As illustrated in Figure 3a, the excitation spectrum detected at 570 nm of YMASG:0.05Ce^3+^ displays a main band with the peak of 455 nm in the blue region and an unobvious one at 345 nm. Corresponding to the parity allowed Ce^3+^: 4*f*-5*d* transitions, the PL spectra present a broadband emission, while the PLE spectra show a wide excitation band as well [16,17,18]. Due to a larger crystal field splitting of the Ce^3+^ 5*d* levels in the silicate garnet, the emission spectrum of this phosphor was redshifted compared to the commercially available phosphor YAG:Ce^3+^. A series of YMASG:xCe^3+^ phosphors were prepared to optimize the doping concentration of Ce^3+^ ion, as displayed in Figure 3b. The emission intensity was firstly enhanced with increasing doping concentration of Ce^3+^ ion, and it then reached a maximum value when the doping concentration of Ce^3+^ ion was 0.05 mol. After that, the emission intensity exhibited a concentration quenching effect due to the electric multipolar interaction or the exchange interaction. Meanwhile, a slight movement of the emission peak to longer wavelengths from 558 nm of x = 0.02 to 582 nm of x = 0.09 was observed with the increase in the doping concentration of Ce^3+^ ion. It is commonly known that the 5*d* energy level is significantly more sensitive to the crystal field than the 4*f* ground state, and the specific crystal field effects of YMASG:xCe^3+^ (x = 0.02–0.09) garnet should be taken into consideration. Based on group theory, the 5*d* level of Ce^3+^ ion splits into five sublevels (denoted as 5*d*_1_, 5*d*_2_, …), as displayed in Figure 3c [19]. The increase in the Ce^3+^ doping concentration distorts the ligand field, leading to the compression of the cube. The strengthened crystal field thus drives the splitting of 5*d*_1_ to a lower energy level and that of 5*d*_2_ to a higher one [19,20]. The emission bands in these samples were broader than that of YAG, which means that greater spectral coverage leads to a higher color rendering index (Ra).

### 3.3. Microstructure and Luminescence Properties of the PiG Sample

**Figure 4:**
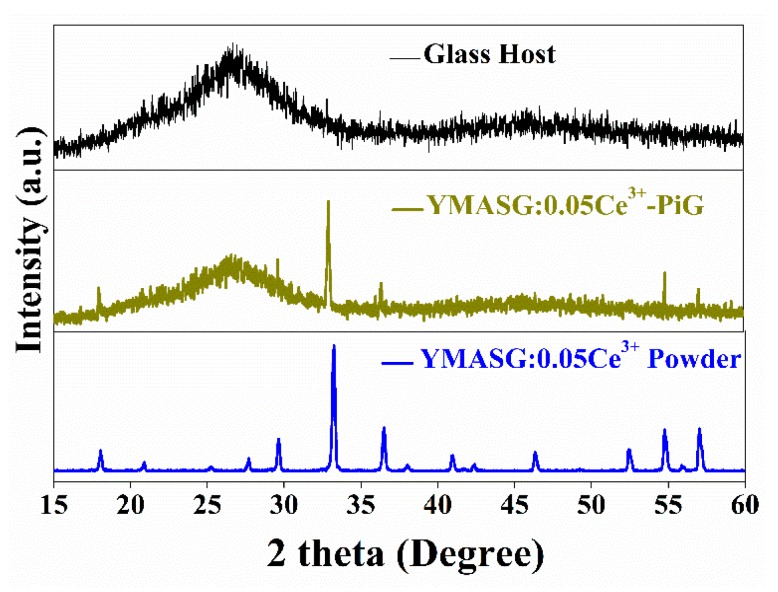
The XRD patterns of YMASG:0.05Ce^3+^ and 8 wt % YMASG:0.05Ce^3+^ PiG composite.

To validate the feasibility of the as-synthesized yellow phosphor YMASG:Ce^3+^ for warm w-LED applications, YMASG:0.05Ce^3+^ was taken as representative and the powder was introduced into the low-melting inorganic precursor glass, forming the PiG color converter. Figure 4 shows the XRD patterns of the glass host, YMASG:0.05Ce^3+^ powder and 8 wt % YMASG:0.05Ce^3+^ single-doped PiG composite, which could mirror the interaction between YMASG:0.05Ce^3+^ and the glass matrix. The main diffraction peaks of the as-prepared PiG sample agree well with those of YMASG:0.05Ce^3+^, suggesting that YMASG:0.05Ce^3+^ was successfully embedded in the glass matrix and no intermediate products were produced by the low-temperature co-sintering method.

**Figure 5:**
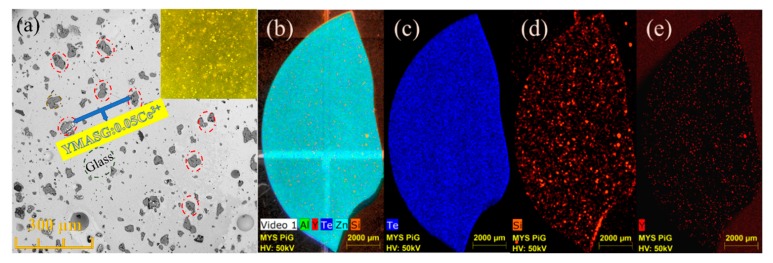
(**a**) The SEM image of the as-prepared PiG sample. (**b**–**e**) The EDS mapping images of the as-prepared PiG sample.

To further investigate the distribution and microstructure of the embedded YMASG:0.05Ce^3+^ particles in the PiG sample, the SEM and EDS mapping images were measured and are displayed in Figure 5. As seen in Figure 5a, no visible aggregations and cracks can be observed, indicating that YMASG:0.05Ce^3+^ particles (red circles) are well distributed in the glass matrix. The inset of Figure 5a presents the optical microscope image of the polished surface of the as-prepared PiG sample under a transmitted white light. Since the glass matrix was almost transparent, the irregularly shaped microcrystalline particles which are randomly located in the glass matrix can be clearly distinguished from glass under the transmitted white light. Subsequently, the EDS mapping of the PiG sample was carried out, as shown in Figure 5b–e. In the EDS mapping images, the elements are represented by different colors, the brighter the color is, the more enriched the element becomes. The Te- and Zn-element portion of the glass matrix was equally distributed due to its high content. In the element distribution maps (Figure 5d,e), the Y and Si elements, which represent the YMASG:0.05Ce^3+^ particles, can be observed in the whole area, indicating that the YMASG:0.05Ce^3+^ crystalline phase is uniformly distribution over the glass matrix. All these results strongly suggest that the YMASG:0.05Ce^3+^ was successfully incorporated into the glass host.

**Figure 6:**
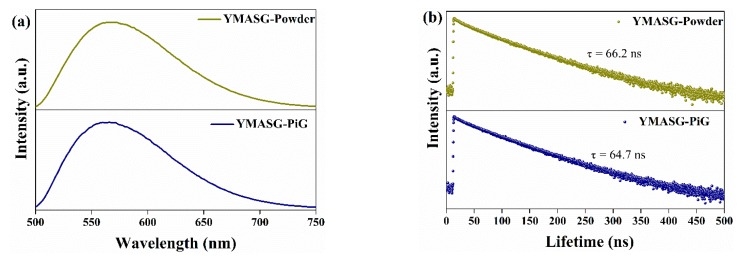
(**a**) The PL spectra of YMASG:0.05Ce^3+^ and YMASG:0.05Ce^3+^ PiG composite under 455 nm light excitation. (**b**) Decay curves of YMASG:0.05Ce^3+^ and YMASG:0.05Ce^3+^ PiG composite.

The PL spectra of YMASG:0.05Ce^3+^ and YMASG:0.05Ce^3+^ PiG composite excited by blue light are illustrated in Figure 6a. As expected, the steady-state PL spectra of YMASG:0.05Ce^3+^ PiG composite and the YMASG:0.05Ce^3+^ proved a typical Ce^3+^: 5*d*→4*f* broadband emission and remained almost unchanged. The luminescence quantum efficiencies of YMASG:0.05Ce^3+^ PiG and YMASG:0.05Ce^3+^ powder were measured respectively, as demonstrated in Figure S1. Meanwhile, the decay behavior of Ce^3+^ ion was also studied, as exhibited in Figure 6b. Evidently, the decay curve of the PiG composite was similar to that of the powder counterpart. Consequently, the luminescent properties of YMASG:0.05Ce^3+^ were retained in the glass host.

**Figure 7:**
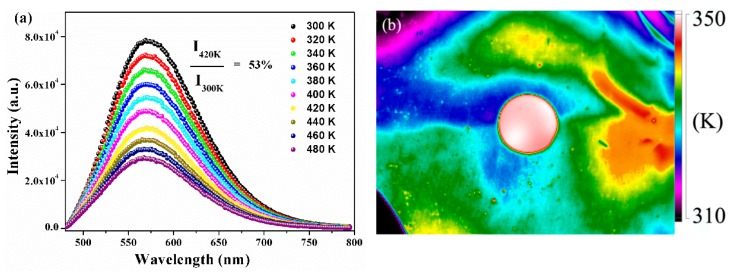
(**a**) Temperature-dependent PL (*λ*_ex_ = 455 nm) spectra of the as-prepared PiG sample. (**b**) Infrared thermal imaging picture of the as-prepared PiG sample.

The thermal stability of encapsulation materials played a significant role for the application of phosphor in the high-power LED field because the LED package would release heat during chip operation. The temperature-dependent PL spectra of as-synthesized YMASG:0.05Ce^3+^–PiG excited at 455 nm are given in Figure 7a. With increasing temperature, the emission intensity gradually decreased and the integrated intensity at 420 K was 53% of the initial intensity at room temperature. The temperature-dependent PL spectra of phosphor particles are shown in Figure S2. As the temperature elevated from 300 to 480 K, the integrated emission intensities gradually declined and the *I_em_* at 440 K dropped to 46% of the initial intensity. Particularly, the PiG sample possessed a slower rate of emission intensity decline compared with that of YMASG:0.05Ce^3+^ due to its relatively high thermal conductivity (0.71 Wm^−1^·K^−1^) to release heat and subsequently reduce the possibility of the Ce^3+^ ion nonradiative transition. Additionally, the weak shift observed in the PL spectra implies that the chromatic shifting of white LEDs will not arise during use. The CIE parameters of the phosphor and the corresponding PiG samples were measured and presented at different temperatures (as shown in Figure S3). The CIE coordinates of the PiG sample just slightly shifted from (0.483, 0.504) to (0.473, 0.511). However, the CIE coordinates of the powder sample were found to be blue-shifted from (0.485, 0.503) to (0.468, 0.512). Tiling the PiG sample on a flat aluminum substrate, which was constantly heated by an electronic hot plate, the infrared thermal imaging picture was taken, and this is shown in Figure 7b. Obviously, although the aluminum plate had a lower temperature than the PiG sample, the PiG sample exhibited a more uniform distribution of temperature, which is beneficial for maintaining the stability of the glass matrix.

### 3.4. The Performance of the PiG-Based LEDs

**Figure 8:**
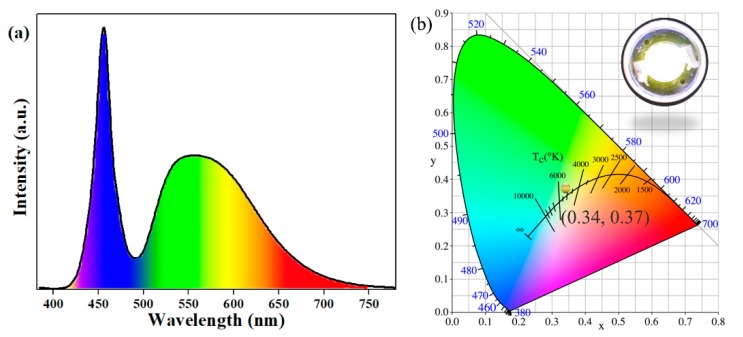
(**a**) The EL spectrum of the PiG-based white LED at an operating current of 350 mA. (**b**) The CIE chromaticity diagram and a digital photograph of the PiG-based LED in operation.

The Ce-concentration-dependent electroluminescence (EL) spectra of PiGs are shown in Figure S4. The emission intensity of the assembled w-LEDs gradually intensified as Ce^3+^ content increased, and then approached a maximum value when the doping concentration of Ce^3+^ was 0.05 mol. After that, the emission intensity decreased. Further combining the developed PiG coating with a commercial 455 nm blue-emitting chip, a white LED device was constructed. The EL spectrum driven by the 350 mA current of this device is illustrated in Figure 8a. The w-LED lamp produced a luminous efficacy of 54.5 lm/W, a CCT of 5274 K, and a CRI value of 79.5. Figure 8b shows the position of the w-LED in the CIE diagram and a digital photograph of the PiG-based white LED under an operating current of 350 mA. It is apparent that the as-prepared device had color coordinates of (0.34, 0.37) and emitted warm white light. To evaluate the application for high-power LED, Figure S5 shows the EL spectra of the fabricated PiG-based w-LEDs under the current regulation (20–500 mA). Generally, the performance of devices was affected by aging temperature. However, after aging for 140 h in 150 °C, no considerable changes in *T*c and *R*a were detected for PiG-based warm w-LED, as exhibited in Figure S6. These results clearly demonstrate that the PiG-based w-LED exhibits excellent heat-resistance performance. In the future, by increasing the amount of phosphors or the PiG thickness, the color temperature of the combined white light is expected to reach 4000~4500 K and satisfy the demand for warm white LED.

## 4. Conclusions

A garnet-based phosphor Y_2_Mg_2_Al_2_Si_2_O_12_:Ce^3+^, which emits strong and broad yellow-orange light, was successfully synthesized via cation substitution. With an increasing doping concentration of Ce^3+^ ions, a larger crystal field splitting of Ce^3+^ 5*d* levels arose, leading to a red-shift in the PL spectra. To evaluate the suitability of YMASG:Ce^3+^ as a warm light converter, YMASG:0.05Ce^3+^ was dispersed directly into the low-melting precursor glass, and the particles were uniformly distributed in glass. Notably, the assembled high-powered w-LED device exhibited a CCT of 5274 K and a CRI of 79.5 when it was driven by a 350 mA current. Consequently, the developed YMASG:Ce^3+^ phosphor and the related PiG are promising candidates for the application in high-powered warm w-LEDs.

## Figures and Tables

**Table 1:** Refined crystallographic data and reliability factors for Y_1.95_Mg_2_Al_2_Si_2_O_12_:0.05Ce^3+^.

Formula	Y_1.95_Mg_2_Al_2_Si_2_O_12_:0.05Ce^3+^
Crystal System	cubic
Space Group	*Ia-3d*
a = b = c/Å	11.94
Volume/Å^3^	1702.1
*R*_p_ (%)	10.03
*R*_wp_ (%)	7.62

## Supplementary Materials

The following are available online at http://www.mdpi.com/1996-1944/11/10/1792/s1, Figure S1: The quantum efficiencies of the phosphor and the corresponding PiG samples; Figure S2: Temperature-dependent PL (λ_ex_ = 455 nm) spectra of the YMASG:0.05Ce^3+^ powder sample; Figure S3: Temperature-dependent CIE coordinates of the PiG and powder samples; Figure S4: Ce-concentration-dependent EL spectra of the PiGs; Figure S5: EL spectra the fabricated PiG-based w-LEDs under the current regulation (20–500 mA).
