# Supplementary material for: Chromaticity-Tunable and Thermal Stable Phosphor-in-Glass Inorganic Color Converter for High Power Warm w-LEDs
Source: Materials (Basel). 2018 Sep 21;11(10):1792. doi: 10.3390/ma11101792 (PMC6213331; doi:10.3390/ma11101792)
Supplement: Supplementary file 1 [file materials-11-01792-s001.pdf]

materials

Supporting Information

Chromaticity-Tunable and Thermal Stable Phosphor-in-Glass Inorganic Color Converter for High Power Warm w-LED

Zikun Chen, Bo Wang *, Xiaoshuang Li, Dayu Huang, Hongyang Sun and Qingguang Zeng *

School of Applied Physics and Materials, Wuyi University, Jiangmen 529020, China; chenzk1993@163.com (Z.C.); lixiaoshuang12@mails.ucas.ac.cn (X.L.); dyhuang@ciac.ac.cn (D.H.); 15667096212@163.com (H.S.)

* Correspondence: wangbo312@mails.ucas.ac.cn (B.W.); zengqg@mail.ustc.edu.cn (Q.Z.)

Received: 12 August 2018; Accepted: 14 September 2018; Published: 21 September 2018

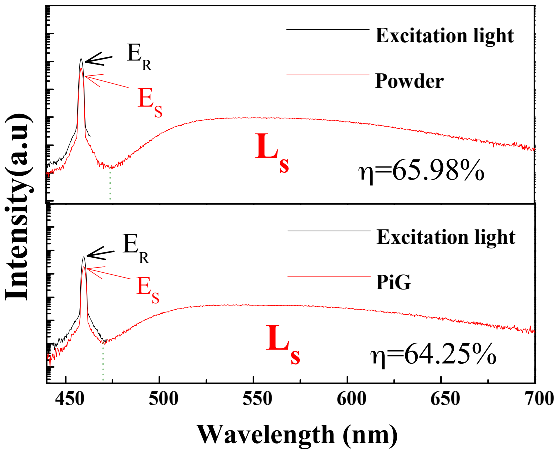

Figure. S1. The quantum efficiencies of the phosphor and the corresponding PiG samples.

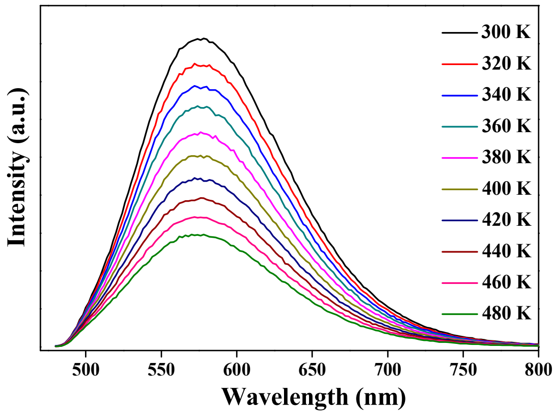

Figure. S2. Temperature dependent the PL (λ_{ex} = 455 nm) spectra of the YMASG:0.05Ce^{3+} powder sample.

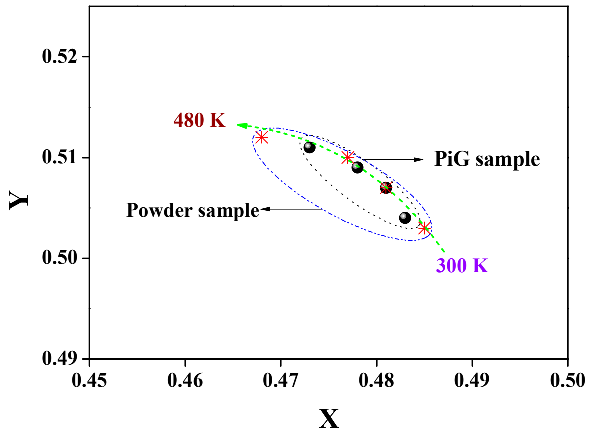

Figure. S3. Temperature-dependent CIE coordinates of the PiG and powder samples.

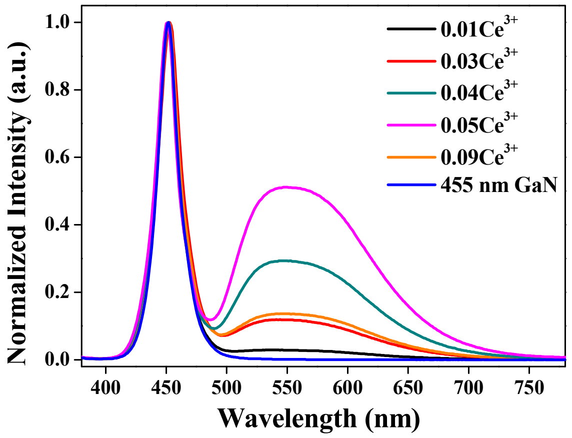

Figure. S4. Ce-concentration dependent EL spectra of the PiGs.

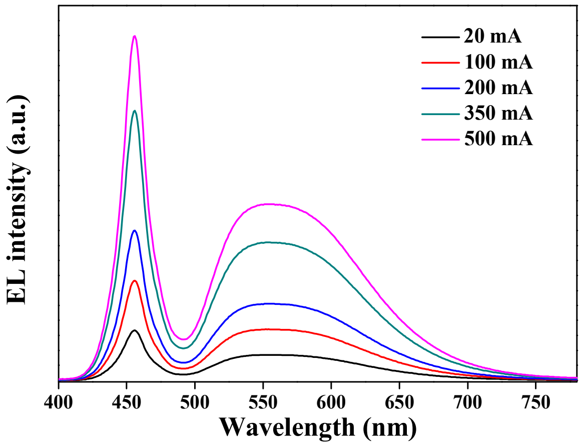

Figure. S5. EL spectra the fabricated PiG-based w-LEDs under the current regulation (20-500 mA).

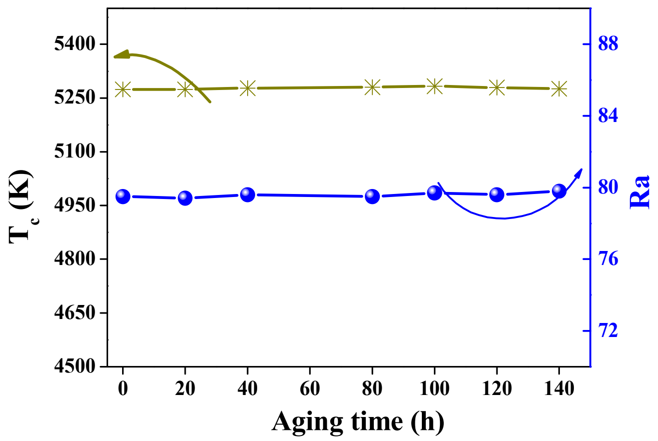

Figure. S6. The variation of T_c and Ra in PiG-based w-LED in the aging process.

© 2018 by the authors. Submitted for possible open access publication under the terms and conditions of the Creative Commons Attribution (CC BY) license (http://creativecommons.org/licenses/by/4.0/).
